# Impact of the 2015 El Nino event on winter air quality in China

**DOI:** 10.1038/srep34275

**Published:** 2016-09-27

**Authors:** Luyu Chang, Jianming Xu, Xuexi Tie, Jianbin Wu

**Affiliations:** 1 Shanghai Meteorological Service, Shanghai, 200030, China; 2 Shanghai Key Laboratory of Meteorology and Health, Shanghai, 200030, China; 3 Key Laboratory of Aerosol Chemistry & Physics, SKLLQG, Institute of Earth Environment, Chinese Academy of Sciences, Xi’an, 710061, China; 4 Center for Excellence in Urban Atmospheric Environment, Institute of Urban Environment, Chinese Academy of Sciences, Xiamen 361021, China; 5 National Center for Atmospheric Research (NCAR), Boulder, 80303, USA

## Abstract

During the winter of 2015, there was a strong El Nino (ENSO) event, resulting in significant anomalies for meteorological conditions in China. Analysis shows that the meteorological conditions in December 2015 (compared to December 2014) had several important anomalies, including the following: (1) the surface southeasterly winds were significantly enhanced in the North China Plain (NCP); (2) the precipitation was increased in the south of eastern China; and (3) the wind speeds were decreased in the middle-north of eastern China, while slightly increased in the south of eastern China. These meteorological anomalies produced important impacts on the aerosol pollution in eastern China. In the NCP region, the PM_2.5_ concentrations were significantly increased, with a maximum increase of 80–100 μg m^−3^. A global chemical/transport model (MOZART-4) was applied to study the individual contribution of the changes in winds and precipitation to PM_2.5_ concentrations. This study suggests that the 2015El Nino event had significant effects on air pollution in eastern China, especially in the NCP region, including the capital city of Beijing, in which aerosol pollution was significantly enhanced in the already heavily polluted capital city of China.

It is well known that the El Nino (ENSO) events can produce significant anomalies in atmospheric general circulations and weather conditions. The ENSO events cause the changes in sea surface temperature (SST) in the Pacific Ocean, impact the Walker Circulation, and displace the convective area. These changes in atmospheric circulation cause anomalies in the monsoon system and moisture fields in eastern Asia^1^. Because there are strong interactions between weather conditions and haze pollutions in eastern China^2^,^3^, it is important to carefully study the weather perturbation during the ENSO events and its impacts on haze pollutions in China, especially in the NCP region, in which the capital city (Beijing) of China is located.

In the last 40 years, the rapid growth of economical development resulted in frequent occurrences of haze pollutions in eastern China, especially in the North China Plain (NCP)^2^,^3^,^4^,^5^. The serious haze pollution events occurred frequently during winter, and were closely related to meteorological conditions^2^,^6^. In the NCP region, these studies demonstrated that wind directions had an important effect on the occurrences of haze events. For example, the heavy haze events often occurred during southerly and southeasterly wind periods, and disappeared during northwesterly wind periods. The detailed explanation for the effect of wind directions on the haze occurrences is showed in the supplemental information of this paper. As a result, the changes in weather conditions attributed to the ENSO events could lead to significant effects on the haze occurrences in the NCP and other regions of China.

In December 2015, there was significant variability of SST over the Middle-East Pacific Ocean. The SST increased more than 2.4 degrees compared with the values in normal years, indicating an occurrence of a strong ENSO event. This strong ENSO event (2015-ENSO) provided an opportunity to study the meteorological anomalies and their effect on air pollutions during this period. In this study, the meteorological and air pollution anomalies between Dec. 2014 and Dec. 2015 were compared, and the impacts of the ENSO on the meteorological parameters and the air quality in eastern China were investigated.

To systemically study these ENSO effects, intensive surface measurements of meteorological data, including wind direction, wind speed, and precipitation at 2,540 monitoring sites in eastern China were used in the analysis. The air pressure, temperature, geo-potential height, and winds from the NCEP/NCAR re-analyzed meteorological data (NCEP-National Centers for Environmental Prediction; NCAR-National Center for Atmospheric Research) were applied to study weather patterns. The surface PM_2.5_ (particle matter with diameter < 2.5 μm) concentrations measured by the Chinese National Environmental Monitoring Center (CNEMC) at 367 monitoring stations were applied for the air pollution study. A state of the art of global chemistry transport model (MOZART-4; Model for Ozone and Related chemical Tracers, version-4)^7^,^8^ was used to study the relative contributions of different meteorological parameters (such as wind and precipitation) to the air quality in eastern China.

## Discussion

**Figure 1:**
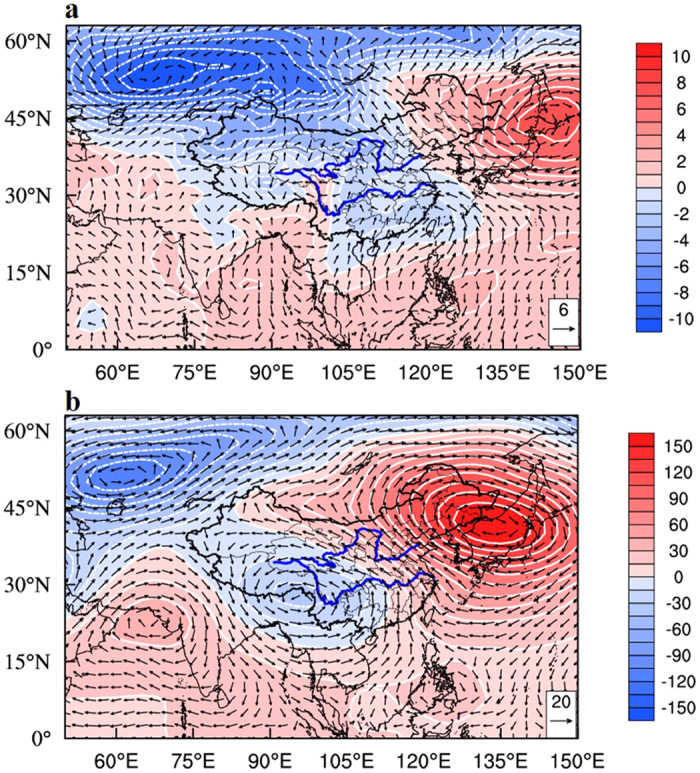
(**a**) Anomalies of monthly mean general circulation between Dec. 2015 and Dec. 2014 in eastern Asia. The shaded and the white lines represent the sea level pressure (hPa) and the vectors for the 10-meter wind field (m s^−1^). NCEP/NCAR re-analysis data were applied in the study. (**b**) Same as (**a**), except at 500 hPa. The Data/image provided by the NOAA/OAR/ESRL PSD, Boulder, Colorado, USA, from their Web site at http://www.esrl.noaa.gov/psd. The map was generated by The NCAR Command Language (Version 6.3.0) [Software]. (2016). Boulder, Colorado: UCAR/NCAR/CISL/TDD. http://dx.doi.org/10.5065/D6WD3XH5.

To understand the impact of the 2015-ENSO on the air pollution in China, we first study the anomalies of weather conditions by comparing the large-scale circulations between Dec. 2015 and Dec. 2014 (i.e., the monthly mean values in 2015 minus the monthly mean values in 2014). Figure 1a shows the anomalies of sea level pressure (hPa) and 10-meter wind speed (m s^−1^). The results show that there were significant positive anomalies of sea level pressure (SLP) in the Aleutian region but negative anomalies of SLP in the Asian continent. For example, the SLP in the Aleutian region was about 4–10 hPa higher in December 2015 than in December 2014. In contrast, the SLP in the Asian continent was about 4–10 hPa lower in December 2015 than in December 2014. As a result, the anomalies of the SLP in the two regions enhanced southeasterly winds nearby the surface in eastern China, especially in the NCP region. As shown in Fig. 1a, the southeasterly winds in the NCP region (32°–40°N, 114°–121°E) during 2015-ENSO were enhanced by 4–5 m s^−1^. Several previous studies suggested that the occurrences of heavy haze events in the NCP (characterized by daily mean concentrations of PM_2.5_ being greater than 100 μg m^−3^) were significantly dependent on the wind directions^2^,^6^. As a result, the enhanced southeasterly winds played important roles in increasing the haze occurrences in the NCP (see Fig. S1 of the supplementary information for detailed explanations).

Figure 1b shows the anomalies of monthly mean geo-potential height and wind field at 500 hPa. It shows that the Ural-Siberian blocking high and Asia trough were weakened during the 2015-ENSO, leading to weaker cold air being transported from Siberia to the south. The weakened Asia trough also produced a stronger cyclonic circulation near the Bay of Bengal and the South China Sea and transported the warm and moist air from the Bay of Bengal and the South China Sea to the continent of South China. As a result, the water vapor flux and precipitation over the continent of South China were enhanced.

**Figure 2:**
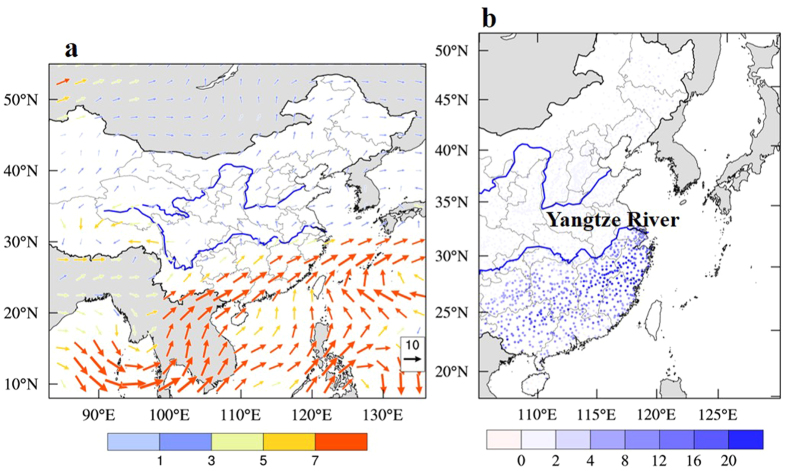
(**a**) Monthly mean anomalies of vertically integrated (1000 hPa to 700 hPa) moisture flux (g m^−1^ s^−1^) between Dec. 2015 and Dec. 2014, and (**b**) anomalies of occurrence frequency (%) of moderate-heavy precipitation (precipitation > 10 mm day^−1^) in eastern Asia. NCEP /NCAR re-analysis data were applied in the study. The Data/image provided by the NOAA/OAR/ESRL PSD, Boulder, Colorado, USA, from their Web site at http://www.esrl.noaa.gov/psd. The map was generated by The NCAR Command Language (Version 6.3.0) [Software]. (2016). Boulder, Colorado: UCAR/NCAR/CISL/TDD. http://dx.doi.org/10.5065/D6WD3XH5.

Figure 2a shows the anomalies of the water vapor flux between Dec. 2015 and Dec. 2014, which were calculated by the vertical integration (1000 hPa to 700 hPa) of moisture flux (g m^−1^ s^−1^), and Fig. 2b shows the anomalies of the frequency of moderate-heavy precipitation (defined as precipitation > 10 mm day^−1^) in China. Two water vapor paths were highlighted during the 2015-ENSO. One originated in the Bay of Bengal and was transported to the continent of South China. Another originated in the South China Sea and was transported to the continent of South China. These two paths converged over the continent of South China, producing higher precipitation in this region (in the south of the Yangtze River) in Dec. 2015 than in Dec. 2014. As shown in Fig. 2b, the frequency of moderate-heavy precipitation significantly increased, with a maximum increase of 15–20% during the 2015-ENSO event. The increased precipitation tended to decrease the PM_2.5_ concentrations due to the particle washout process^9^,^10^.

**Figure 3:**
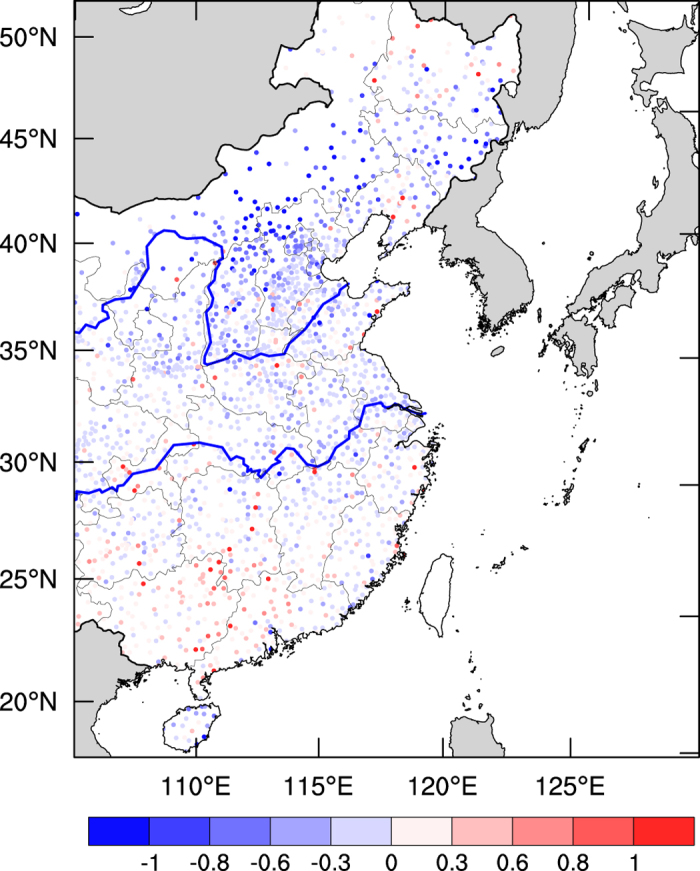
Monthly mean anomalies between Dec. 2015 and Dec. 2014 of surface wind speed (m s^−1^) in eastern China. Rainy days were excluded. The wind speeds were measured by the National Climate Center of China at 2,540 monitoring sites. The map was generated by The NCAR Command Language (Version 6.3.0) [Software]. (2016). Boulder, Colorado: UCAR/NCAR/CISL/TDD. http://dx.doi.org/10.5065/D6WD3XH5.

Another important factor is wind speed, which has an important effect on the PM_2.5_ concentrations^3^,^11^,^12^. Figure 3 shows the anomalies of monthly mean surface wind speeds (m s^−1^) during the 2015-ENSO. It shows that the surface wind speeds were generally reduced in eastern China, especially in the NCP region (north of the Yellow River), with a decrease in surface wind speeds of 0.5–1.0 m s^−1^. In the middle of eastern China (between the Yellow and the Yangtze Rivers), the surface wind speeds were generally decreased, with a maximum reduction of 0.5 m s^−1^. In the south of eastern China (south of the Yangtze River), the changes in surface wind speeds were mixed. In most areas, the surface wind speeds were slightly increased, with a maximum increase of 0.8 m s^−1^. However, there was a small area where the dominant changes were the decreases in wind speeds. The overall changes in the surface wind speeds in this region ranged between −0.6 and +0.8 m s^−1^.

**Figure 4:**
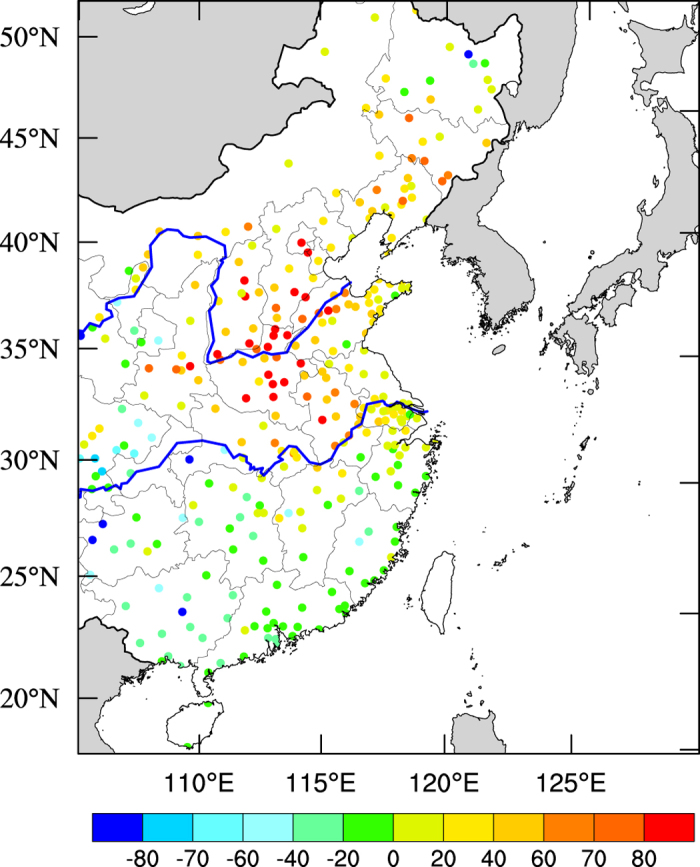
Monthly mean anomalies of measured PM_2.5_ concentrations (μg m^−3^) between Dec. 2015 and Dec. 2014 in eastern China. The PM_2.5_ concentrations were measured by the Chinese National Environmental Monitoring Center (CNEMC) at 367 monitoring stations. The map was generated by The NCAR Command Language (Version 6.3.0) [Software]. (2016). Boulder, Colorado: UCAR/NCAR/CISL/TDD. http://dx.doi.org/10.5065/D6WD3XH5.

Figure 4 shows the anomalies of measured PM_2.5_ concentrations (μg m^−3^) during the 2015-ENSO. The results show that the PM_2.5_ anomalies existed in a complicated spatial distribution. The PM_2.5_ concentrations were significantly increased in the NCP region, with a maximum increase of 80–100 μg m^−3^. In this region, the southeasterly wind direction was enhanced, which was favorable for the increase in the PM_2.5_ concentrations (μg m^−3^). As explained in the supplementary information, the southeasterly wind had two important effects on the PM_2.5_ concentrations in the NCP region. First, the southeasterly wind enhanced the horizontal transport from high emission regions to the NCP region, resulting in an increase in PM_2.5_ concentrations. Second, there are mountains to the west and on the north sides of the NCP region. The pollution plumes transported from the southeastern region were ‘blocked’ by the mountains, leading to an accumulation of PM_2.5_ concentrations in the northwestern area of the NCP region. Another factor that increased the PM_2.5_ concentrations was the decrease in wind speed, which caused the accumulation of PM_2.5_ concentrations in the region.

In the middle of eastern China (between the Yellow and Yangtze Rivers), the PM_2.5_ concentrations were also significantly increased, ranging from 20–80 μg m^−3^, which was coherent with the decrease in the surface wind speed.

In the south of eastern China, the anomalies of PM_2.5_ concentrations had a complex horizontal distribution. In the northeastern corner of the region, there was a slight increase in the PM_2.5_ concentrations (20 μg m^−3^), while there was a general decrease in PM_2.5_ concentrations in most areas of the south of eastern China, which was consistent with the changes in the wind speed in the region. In addition, the enhancement in the precipitation during the 2015-ENSO also contributed to the decrease in PM_2.5_ concentrations in the region.

**Figure 5:**
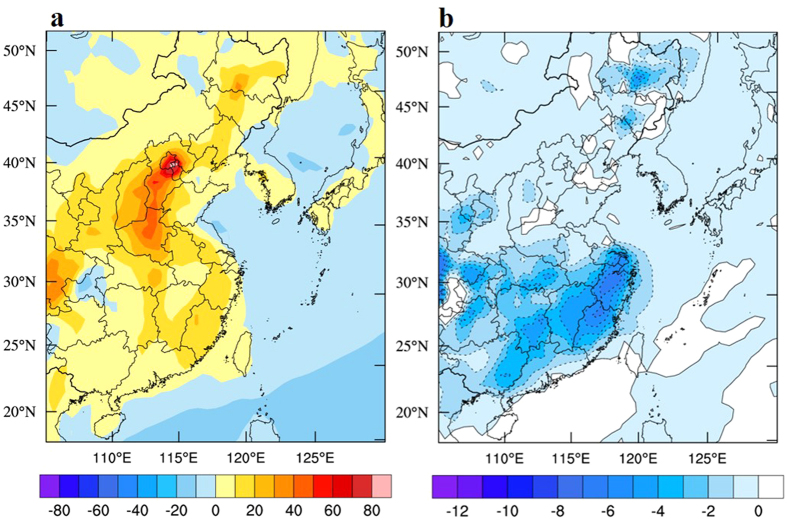
(**a**) The calculated changes in PM_2.5_ concentrations (μg m^−3^) in eastern China without wet deposition of aerosol particles. (**b**) The calculated change in PM_2.5_ concentrations (μg m^−3^) due solely to wet deposition of aerosol particles, the dash line denotes negative change in in PM_2.5_ concentrations. The map was generated by The NCAR Command Language (Version 6.3.0) [Software]. (2016). Boulder, Colorado: UCAR/NCAR/CISL/TDD. http://dx.doi.org/10.5065/D6WD3XH5.

In order to better understand the individual contributions of wind and precipitation to the PM_2.5_ concentrations during the 2015-ENSO, a global chemical transport model (MOZART-4) was applied in this study. The detailed model description is shown in the supplementary information. Figure 5a shows the calculated change in PM_2.5_ concentrations (μg m^−3^) in eastern China without wet deposition of aerosol particles (left panel), and Fig. 5b shows the change in PM_2.5_ concentrations (μg m^−3^) due solely to the wet deposition of aerosol particles (right panel).

The results show that due to the wind effects (with both wind direction and wind speed, without precipitation effects), the PM_2.5_ concentrations were generally higher in eastern China during the 2015-ENSO (Shown in Fig. 5a). The highest concentrations occurred in the NCP region, with a maximum increase of 80–100 μg m^−3^, which was consistent with the measured anomalies of the PM_2.5_ concentrations (see Fig. 4). As explained in the supplementary information, the enhancement of the southeasterly wind played an important role in the increase in PM_2.5_ concentrations. In addition, the decreased wind speeds (0.5–1.0 m s^−1^) also caused an increase in the PM_2.5_ concentrations in the region. In the middle of eastern China, the calculated PM_2.5_ concentrations were modestly increased, ranging from 10 to 50 μg m^−3^. These calculations were consistent with the measured anomalies of the PM_2.5_ concentrations during the 2015-ENSO, suggesting that the decrease in wind speed played an important role in enhancing PM_2.5_ concentrations in the middle-north of eastern China. However, in the south of eastern China, the calculated PM_2.5_ concentrations were increased in most areas, ranging from 0 to 20 μg m^−3^. In contrast, the measured PM_2.5_ concentrations were decreased in most areas. Because there was no wet deposition of aerosol particles in the calculation, the enhanced wet deposition could be important for the decrease in PM_2.5_ concentrations in the south of eastern China. Figure 5b shows the calculated changes in PM_2.5_ concentrations due solely to the wet deposition of aerosol particles. The results show that the enhanced wet deposition (due to the increase in precipitation) produced an important decrease in PM_2.5_ concentrations in the south of eastern China, ranging from 2 to 10 μg m^−3^. The results slightly underestimated the measured decrease in PM_2.5_ concentrations, which suggests that the wet deposition played more important roles than the model predicted.

## Additional Information

**How to cite this article**: Chang, L. *et al*. Impact of the 2015 El Nino event on winter air quality in China. *Sci. Rep.* **6**, 34275; doi: 10.1038/srep34275 (2016).

## Supplementary Material

Supplementary Information
