# Supplementary material for: Impact of the 2015 El Nino event on winter air quality in China
Source: Sci Rep. 2016 Sep 27;6:34275. doi: 10.1038/srep34275 (PMC5037463; doi:10.1038/srep34275)
Supplement: Supplementary Information [file srep34275-s1.doc]

**Supplementary Information for**

**Impact of the 2015 El Nino event on winter air quality in China**

Luyu Chang^1,2^, Jianming Xu^1,2*^, Xuexi Tie^3,4,5*^，Jianbin Wu^1,2^

^1^Shanghai Meteorological Service, Shanghai, 200030, China

^2^Shanghai Key Laboratory of Meteorology and Health, Shanghai, 200030, China

^3^Key Laboratory of Aerosol Chemistry & Physics, SKLLQG, Institute of Earth Environment, Chinese Academy of Sciences, Xi’an, 710061, China

**^4^Center for Excellence in Urban Atmospheric Environment, Institute of Urban Environment, Chinese Academy of Sciences, Xiamen 361021, China**

^5^National Center for Atmospheric Research (NCAR), Boulder, 80303, USA.

**(1) Measurements and data**

**1.1 Measured surface PM_2.5_ concentrations.**

Hourly averaged PM_2.5_ concentrations in 2014 and 2015 were measured by the Chinese National Environmental Monitoring Center (CNEMC), operated by the Ministry of Environmental Protection of the People’s Republic of China (MEPC), at 367 monitoring stations ([http://106.37.208.233:20035](http://106.37.208.233:20035/)). Daily average PM_2.5_ concentrations were calculated from the hourly data. The monthly PM_2.5_ averages were then obtained from the daily averaged concentrations for Dec. 2014 and Dec. 2015.

**1.2 Meteorological data.**

Meteorological data were obtained from two sources: 1) Surface meteorological parameters at 2,540 Chinese monitoring sites were obtained from the National Climate Center of China. The surface wind and daily precipitation were used in this study; 2) General circulation data were obtained from the National Center for Environmental Prediction (NCEP) and National Center for Atmospheric Research (NCAR) reanalysis data (defined as NCEP/NCAR data)^1^. The data have a horizontal resolution of 2.5º×2.5º. Sea level pressure (SLP), geo-potential height, wind speed and direction, and specific humidity data were used in this study.

**(2) Chemical dynamical models**

**2.1 Global chemistry transport model (Mozart-4).**

A global chemistry transport model (MOZART-4; Model for Ozone and Related chemical Tracers, version-4) was used in this study. The detailed model description is shown by Emmons et al. (Ref. 2), and the detailed aerosol modules are shown by Tie et al. (Ref. 3). The MOZART-4 model is a global chemical transport model. The model is designed to study the global distributions of tropospheric trace gases and aerosol particles. In this study, the horizontal resolution of the model is 0.7º×0.7º, with 42 vertical levels. The advective scheme for chemical species used in MOZART is a progenitor called the Model of Atmospheric Transport and Chemistry (MATCH) developed by Rasch et al. (Ref. 4). It uses the flux form semi-Lagrangian transport algorithm of Lin and Rood (Ref. 5), which can be easily and efficiently implemented on the sphere. The deep convection scheme developed by Zhang and McFarlane (Ref. 6) is included in the model. The wet deposition scheme used in MOZART was developed by Brasseur et al. (Ref. 7) considering a first-order loss process. Presently, in-cloud scavenging is formulated for all soluble species (CH_3_OOH, C_3_H_7_OOH, C_3_H_6_OHOOH, CH_3_COCH_2_OOH, CH_3_COOOH, C_2_H_5_OOH, HO_2_NO_2_, ONIT, CH_2_O), except nitric acid and hydrogen peroxide, by the parameterization of Giorgi and Chameides (Ref. 8). The model transport is driven by the European Centre for Medium-Range Weather Forecasts (ECMWF) assimilated wind fields, with 0.5º×0.5º horizontal resolution^9^. The meteorological data are interpolated to fit the model horizontal resolution by using a bilinear interpolation method. In order to study the individual contributions of wind and precipitation to the calculated PM_2.5_ concentrations, two numerical experiments were designed in this study. One was to exclude the effect of wet deposition of aerosol particles, while another was to include the effect of wet deposition of aerosol particles.

**2.2 Regional chemical dynamical model (WRF-Chem)**

A regional chemical/dynamical model (Weather Research and Forecasting, with chemistry; WRF-Chem) was used in this study. The Weather Research and Forecasting (WRF) Model is a mesoscale numerical weather prediction system designed to serve both operational forecasting and atmospheric research needs. The WRF model is a fully compressible and Euler non-hydrostatic model. It calculates winds (u, v, and w), perturbation potential temperature, perturbation geo-potential, and perturbation surface pressure of dry air. It also can optionally output other variables, including turbulent kinetic energy, water vapor mixing ratio, rain/snow mixing ratio, and cloud water/ice mixing ratio. The model physics include bulk scheme, sophisticated mixed-phase physics for cloud-resolving modeling, multi-layer land surface models ranging from a simple thermal model to full vegetation and soil moisture models, including snow cover and sea ice, turbulent kinetic energy prediction or non-local K schemes for planetary boundary layer calculation, and long-wave and short-wave schemes with multiple spectral ands and a simple shortwave scheme.

In addition to the dynamical calculation, a chemical model is on-line coupled with the WRF model (WRF-Chem). A detailed description of WRF-Chem is given by Grell et al. (Ref. 10). The version of the model, as used in the present study, includes on-line calculation of dynamical inputs (winds, temperature, boundary layer, clouds, etc.), transport (advective, convective, and diffusive), dry deposition^11^, gas phase chemistry, radiation and photolysis rates^12,13^, and surface emissions (including on-line calculation of biogenic emission). A positive-definite scheme was applied to calculate the advection of the chemistry variables. The transport scheme well conserves scalar mass locally and globally and is consistent with the WRF-ARW mass conservation equation. The ozone formation chemistry is represented in the model by the RADM2 (Regional Acid Deposition Model, version 2) gas phase chemical mechanism^14^, which includes 158 reactions among 36 species. A full aerosol particle formation is also included in the model.

In this study, the model resolution is 3x3 km in the horizontal direction, in a 600 x 600 km domain centered in NCP. The model has 31 vertical levels, non-uniformly spaced, from the surface to 20 mb. The lateral boundary and initial conditions of meteorological inputs (winds, temperature, etc.) are constrained with National Center for Environmental Prediction (NCEP) data. The initial chemical conditions are constrained by the results of a global chemical transport model (MOZART-4). The model calculations with two different cases were conducted. One was under a typical southerly/southeasterly wind condition, calculating the PM_2.5_ distribution from 8 to 9 October 2014, while another was under a typical northerly wind condition, calculating the PM_2.5_ distribution from 28 to 29 October 2013.

**(3) Effect of southeasterly winds on PM_2.5_ in the NCP.**

In order to demonstrate the effect of the enhanced southeasterly wind on the PM_2.5_ concentrations in the NCP region, we used the WRF-Chem model to simulate the PM_2.5_ concentrations under a typical southerly/southeasterly wind condition. Figure S1 shows the topography of the NCP region. There are two mountains in the north and west of the NCP. The Yanshan Mountains are located in the north of the NCP, with an east-west direction, and the Taihang Mountains are located in the west of the NCP, with a southwest-northeast direction (see the left panel of Fig. S1). Figure S1 also shows the spatial distribution of PM_2.5_ emissions (see the right panel of Fig. S1). It shows that, in the northwestern area of Beijing (the capital city of China located in the NCP), the topography is covered by mountains and grasslands, with a very small population. In this case, the northwesterly wind transports relatively clean air to the NCP. In contrast, in the south of Beijing (the main area of the NCP), the population is very dense, with several mega/large cities and high PM_2.5_ emissions. As a result, the southerly/southeasterly wind transports polluted air to the northern area of the NCP. These two major factors (the spatial distributions of the mountains and the PM_2.5_ emissions) have an important effect on the spatial distributions of PM_2.5_ concentrations in the NCP.


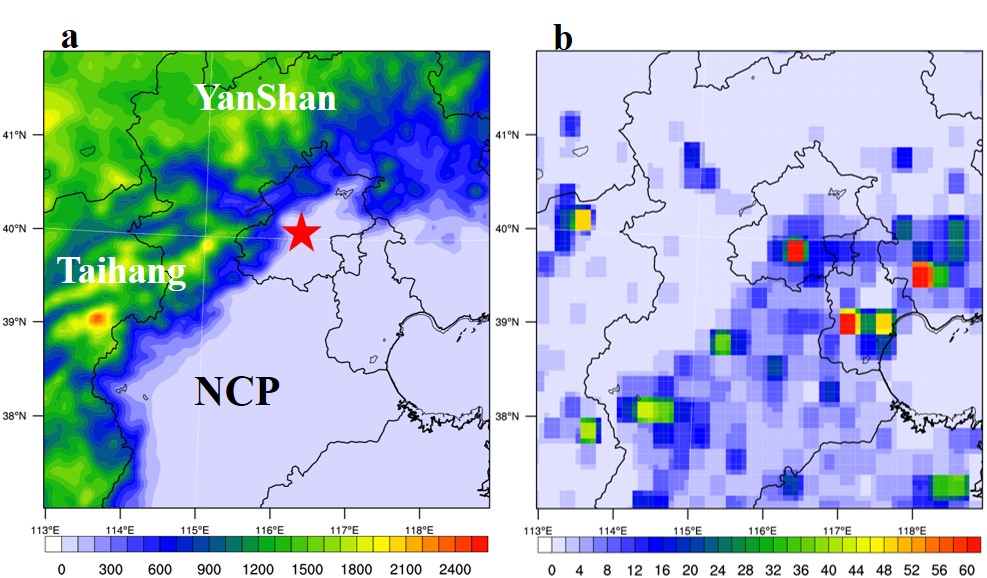


**Figure S1. The spatial distributions of (a) the topography (m) and (b) the PM_2.5_ emissions (g s^-1^) in the NCP region. The red shows Beijing City (the capital city of China). There are two mountains in the north and west of the NCP. The Yanshan Mountains are located in the north of the NCP, with an east-west direction, and the Taihang Mountains are located in the west of the NCP with a southwest-northeast direction (see the left panel of Fig. S1). The spatial distribution of PM_2.5_ emissions (see the right panel of Fig. S1) shows that in the northwestern area of Beijing, the topography is covered by mountains and grasslands, with a very small population. As a result, the northwesterly wind transports relatively clean air to the NCP. In contrast, in the south of Beijing (the main area of the NCP), the population is very dense, with several mega/large cities and high PM_2.5_ emissions. In this case, the southerly/southeasterly wind transports polluted air to the northern area of the NCP. The map was generated by The NCAR Command Language (Version 6.3.0) [Software]. (2016). Boulder, Colorado: UCAR/NCAR/CISL/TDD.** [**http://dx.doi.org/10.5065/D6WD3XH5**](http://dx.doi.org/10.5065/D6WD3XH5)**.**

Figure S2 shows the calculated PM_2.5_ concentrations under a typical southerly/southeasterly wind condition. In this case, the calculated PM_2.5_ concentrations were high, with a maximum concentration of 250 μg m^-3^. There was also an indication that the PM_2.5_ concentrations accumulated along the foothill of the Taihang and Yanshan Mountains. As explained by previous studies^12^, the mountains and the high PM_2.5_ emissions located in the middle-south of the NCP play important roles in the PM_2.5_ accumulation along the foothill of the mountains. First, the high PM_2.5_ concentrations in the middle-south area of the NCP (with high PM_2.5_ emissions) were transported from the south to the north of the NCP. Second, high PM_2.5_ concentrations accumulated along the foothill of the mountains. This is because the wind speeds were reduced along the foothill of the mountains, and the high PM_2.5_ concentrations were blocked by the mountains, resulting in the PM_2.5_ accumulation along the foothills of the Taihang and Yanshan Mountains.


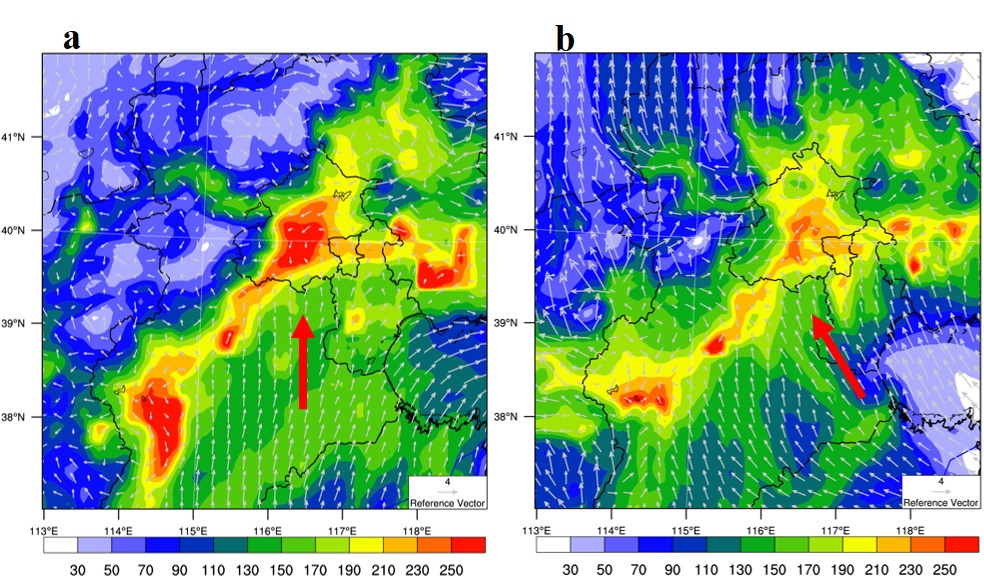


**Figure S2. The calculated PM_2.5_ concentrations (**μg m^-3^**) under a typical southerly/southeasterly wind condition at (a) 08:00 (LST), October 8, 2014 and (b) 23:00 (LST), October 9, 2014. The shade denotes PM_2.5_ concentration. The gray arrows denote 10m-winds. The red arrows denote dominant wind direction. The result shows that the PM_2.5_ concentrations accumulated along the foothill of the Taihang and Yanshan Mountains because of 2 main reasons. First, the high PM_2.5_ concentrations in the middle-south area of the NCP (with high PM_2.5_ emissions) were transported from the south to the north of the NCP. Second, the high PM_2.5_ concentrations were blocked by the mountains, resulting in PM_2.5_ accumulation along the foothills of the Taihang and Yanshan Mountains. The map was generated by The NCAR Command Language (Version 6.3.0) [Software]. (2016). Boulder, Colorado: UCAR/NCAR/CISL/TDD.** [**http://dx.doi.org/10.5065/D6WD3XH5**](http://dx.doi.org/10.5065/D6WD3XH5)**.**

In contrast, Figure S3 shows the PM_2.5_ concentrations under a typical northerly wind condition. The result shows that the high PM_2.5_ concentrations were rapidly dispersed, and the PM_2.5_ concentrations were significantly decreased, especially along the foothills of the mountains. This study illustrates that the wind directions play important roles in controlling the PM_2.5_ concentrations, especially along the foothills of the mountains in the NCP. This result demonstrated that the enhancement of the southerly or southeasterly winds during the 2015-ENSO can significantly increase the PM_2.5_ concentrations in the NCP region.


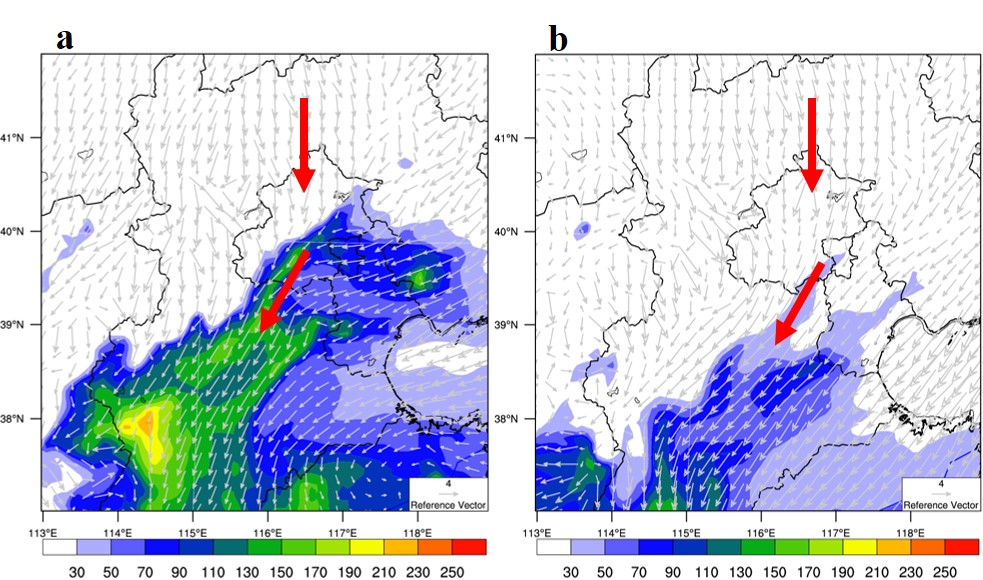


**Figure S3. The calculated PM_2.5_ concentrations (μg m^-3^) under a typical northerly wind condition at (a) 22:00 (LST) October 28, 2013 and (b) 05:00 (LST) October 29, 2013. The shade denotes PM_2.5_ concentration. The gray arrows denote 10m-winds. The red arrows denote dominant wind direction. The result shows that the high PM_2.5_ concentrations were rapidly dispersed, and the PM_2.5_ concentrations were significantly decreased, especially along the foothills of the mountains. This study illustrates that the wind directions play important roles in controlling the PM_2.5_ concentrations, especially along the foothills of the mountains in the NCP. The map was generated by The NCAR Command Language (Version 6.3.0) [Software]. (2016). Boulder, Colorado: UCAR/NCAR/CISL/TDD.** [**http://dx.doi.org/10.5065/D6WD3XH5**](http://dx.doi.org/10.5065/D6WD3XH5)**.**

**Reference**

1. Kalnay, E., et al. The NCEP/NCAR 40-year reanalysis project. *Bulletin of the American Meteorological Society* **77**, 437-471 (1996).
2. Emmons, L.S. et al. Description and evaluation of the Model for Ozone and Related chemical Tracers, version4 (MOZART-4). *Geoscientific Model Development* **3**, 43-67 (2010).
3. Tie, X., et al. Assessment of the global impact of aerosols on tropospheric oxidants. *J. Geophys. Res.* **110** (D03204), doi:10.1029/2004JD005359 (2005).
4. Rasch, P. J., Mahowald, N. M. & Eaton, B. E. Representations of transport, convection, and the hydrologic cycle in chemical transport models: Implications for the modeling of short-lived and soluble species. *J. Geophys. Res.* 102, 28127–28138 (1997).
5. Lin, S.J. & Rood, R.B. Multidimensional flux-form semi-Lagrangian transport schemes*. Mon. Wea. Rev.* **124***,* 2046-2070 (1996).
6. Zhang, G.J. & McFarlane N.A. Sensitivity of climate simulations to the parameterization of cumulus convection in the Canadian Climate Center General circulation model. *Atmos. Ocean.* **33**, 407-446 (1995).
7. Brasseur, B. et al. MOZART: A global chemical transport model for ozone and related chemical tracers, Part 1: Model description. J. Geophys. Res. 103, 28265-28289 (1998).
8. Giorgi, F. & Chameides W.L. The rainout parameterization in a photochemical model. J. Geophys. Res. 90, 7872-7880 (1985).
9. Simmons, A.J., Uppala, S.M., Dee, D.P. & Kobayashi, S. ERA-Interim: New ECWMF reanalysis products from 1989 onwards. *ECWMF Newsletter.* **110**, 25-35 (2007).
10. Grell, G. A. et al. Fully coupled "online" chemistry within the WRF model. *Atmos. Environ.* **39,** 6957-6975 (2005).
11. Wesely, M.L. Parameterization of surface resistance to gaseous dry deposition in regional-scale numerical models. *Atmos. Environ.* ***23*,** 1293-1304 (1989).
12. Madronich, S. & Flocke, S. The role of solar radiation in atmospheric chemistry, in *Handbook of Environmental Chemistry* (P. Boule, ed.), Springer-Verlag, Heidelberg, pp. 1-26 (1999).
13. Tie, X., Madronich, S., Walters, S., Pasch, P. & Collins, W. Effect of Clouds on photolysis and oxidants in the troposphere. *J. Geophys. Res.* **108***,* 4642, doi: 10.1029/2003JD003659 (2003).
14. Chang, J.S. et al. The regional acid deposition model and engineering model, *State-of-Science/Technology, Report 4, National Acid Precipitation Assessment Program,* Washington D.C. (1989).
15. Zhao, S.Y., Tie, X.X., Cao, J.J. & Zhang, Q. Impacts of mountains on black carbon aerosol under different synoptic meteorology conditions in the Guanzhong region, China. *Atmos. Res*. **164-165(1)**, 286-296 (2015).
